# *RASSF1A* promoter methylation in high-grade serous ovarian cancer: A direct comparison study in primary tumors, adjacent morphologically tumor cell-free tissues and paired circulating tumor DNA

**DOI:** 10.18632/oncotarget.15249

**Published:** 2017-02-10

**Authors:** Lydia Giannopoulou, Issam Chebouti, Kitty Pavlakis, Sabine Kasimir-Bauer, Evi S. Lianidou

**Affiliations:** ^1^ Analysis of Circulating Tumor Cells Laboratory, Laboratory of Analytical Chemistry, Department of Chemistry, University of Athens, University Campus, Athens, 15771, Greece; ^2^ Department of Gynecology and Obstetrics, University Hospital of Essen, University of Duisburg-Essen, Essen, D-45122, Germany; ^3^ Pathology Department, IASO Women's Hospital, Marousi, Athens, 15123, Greece

**Keywords:** circulating tumor DNA, RASSF1A, high-grade serous ovarian cancer, methylation specific PCR, high resolution melting analysis

## Abstract

The *RASSF1A* promoter is frequently methylated in high-grade serous ovarian cancer (HGSC). We examined *RASSF1A* promoter methylation in primary tumors, adjacent morphologically tumor cell-free tissues and corresponding circulating tumor DNA (ctDNA) samples of patients with HGSC, using a real-time methylation specific PCR (real-time MSP) and a methylation-sensitive high-resolution melting analysis (MS-HRMA) assay for the detection and semi-quantitative estimation of methylation, respectively. Two groups of primary HGSC tumor FFPE samples were recruited (Group A n=67 and Group B n=61), along with matched adjacent morphologically tumor cell-free tissues (n=58) and corresponding plasma samples (n=59) for group B. Using both assays, *RASSF1A* promoter was found highly methylated in primary tumors of both groups, and at lower percentages in the adjacent morphologically tumor cell-free tissues. Interestingly, *RASSF1A* promoter methylation was also observed in ctDNA by real-time MSP. Overall survival (OS) was significantly associated with *RASSF1A* promoter methylation in primary tumor samples using MS-HRMA (P=0.023). Our results clearly indicate that *RASSF1A* promoter is methylated in adjacent tissue surrounding the tumor in HGSC patients. We report for the first time that *RASSF1A* promoter methylation provides significant prognostic information in HGSC patients.

## INTRODUCTION

Ovarian cancer represents the third most frequent gynecological cancer and the fifth leading cause of cancer-related death in women [1]. Epithelial ovarian cancer is the main type, characterized by histological and molecular heterogeneity. The most common subtype, high-grade serous ovarian cancer (HGSC), is often diagnosed at an advanced stage and little progress has been achieved in standard treatment and overall survival (OS) during the last three decades [2]. Primary disease is treated with surgical removal of the tumor, followed by a combination of platinum and taxane-based chemotherapy [3, 4] with about 20% of patients found to be resistant to this treatment [5, 6]. New multimodal therapeutic concepts now include targeted therapy applying Bevacizumab or the PARP inhibitor Olaparib in certain clinical situations [7, 8].

It is now clear that epigenetic alterations hold an important role in cancer initiation and progression and that aberrant DNA methylation, especially promoter hypermethylation of tumor suppressor genes is a frequent event in most human cancers [9]. Epigenetic inactivation of a tumor suppressor gene often results from its promoter methylation and is considered as an early event during carcinogenesis [10]. Many studies have reported methylation changes in epithelial ovarian cancer [11] and a recent review summarizes the differences in the observed methylation patterns in the main histological subtypes of the disease, including HGSC [12].

Cell-free DNA (cfDNA) circulates at high concentrations in cancer patients and can be used for the detection of several molecular alterations related to cancer development [13]. Circulating tumor DNA (ctDNA) is a small percentage of cfDNA that is shed in circulation by tumor cells and carries all these molecular alterations including tumor specific mutations, microsatellite instability (MI) [13], loss of heterozygosity (LOH) [14], and DNA methylation [15]. Circulating tumor DNA is a very promising non-invasive diagnostic, prognostic and predictive tool, since it provides an easily accessible source of DNA derived from the tumor [16]. Our group has reported *SOX17* [17, 18], *CST6* [19] and *BRMS1* [20] promoter methylation in cfDNA in breast and non-small cell lung cancer patients.

The *RASSF1* gene belongs to the Ras-association domain family that consists of ten members. RASSF proteins contribute to microtubule stability and they are involved in cell cycle regulation, apoptosis, cell migration and cell adhesion. The *RASSF1* gene is found on the 3p21.3 locus and comprises eight exons. Its two promoter regions and the implied alternative splicing are responsible for the eight isoforms A-H. *RASSF1A* and *RASSF1C* are mostly studied so far, especially *RASSF1A* gene isoform that definitely acts as a tumor suppressor in human cancer [21, 22]. *RASSF1A* is involved in molecular pathways including Ras/PI3K/AKT, Ras/RAF/MEK/ERK, Hippo pathways and β-catenin signaling pathway [22, 23]. The *RASSF1A* gene is frequently inactivated by aberrant promoter hypermethylation in the majority of human malignancies, including breast, lung, gastrointestinal, bladder, head and neck cancer and gynecological cancers, endometrial and cervical cancer [23]. In ovarian cancer, *RASSF1A* promoter methylation has been identified in many studies [24], but no significant association with clinical outcome has been reported so far.

The aim of the present study was to examine the prognostic significance of *RASSF1A* promoter methylation in primary tumors, matched adjacent morphologically tumor cell-free tissues surrounding the tumor and the corresponding plasma samples of patients with HGSC. To evaluate the clinical significance of *RASSF1A* promoter methylation in HGSC, we applied a highly sensitive real-time methylation specific PCR (real-time MSP) assay [25] for the detection of *RASSF1A* promoter methylation and compared it to a methylation-sensitive high-resolution melting analysis (MS-HRMA) assay. We further directly compared *RASSF1A* promoter methylation between primary tumors, matched adjacent tissues and corresponding plasma ctDNA. To the best of our knowledge, this is the first study on the evaluation of *RASSF1A* promoter methylation status in HGSC that is based on matched primary tumors, adjacent tissues and corresponding plasma samples from the same patients. Our results clearly indicate that the *RASSF1A* promoter is methylated in adjacent tissue surrounding the tumor in HGSC patients. We also report for the first time that *RASSF1A* promoter methylation provides significant prognostic information in HGSC patients.

## RESULTS

**Figure 1:**
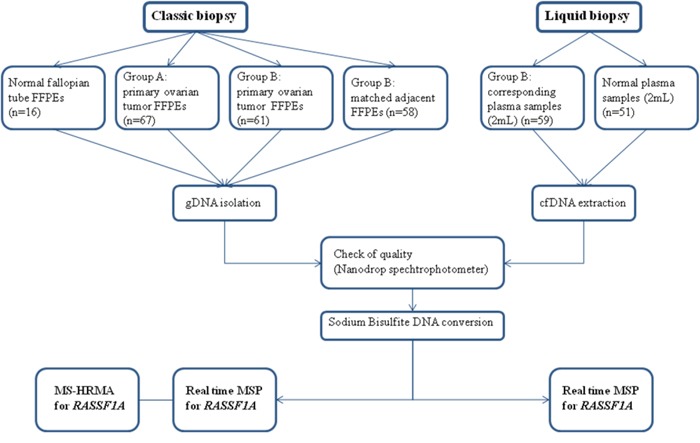
A schematic diagram of our study

A schematic diagram of our study is shown in Figure 1.

### *RASSF1A* promoter methylation status in HGSC by real-time MSP

*RASSF1A* promoter methylation status was first evaluated in the group A by real-time MSP. According to our results, *RASSF1A* promoter was methylated in 27/67 (40.3%) primary tumor samples. *RASSF1A* promoter methylation status was further evaluated in the group B. According to our results, *RASSF1A* promoter was methylated in 25/61 (41.0%) primary tumor samples. In the group of adjacent morphologically tumor cell-free tissues of group B, 17/58 (29.3%) samples were found methylated. In cfDNA, isolated from corresponding plasma, 15/59 (25.4%) samples were found positive for *RASSF1A* promoter methylation.

### Semi-quantitative estimation of *RASSF1A* promoter methylation by MS-HRMA

**Figure 2:**
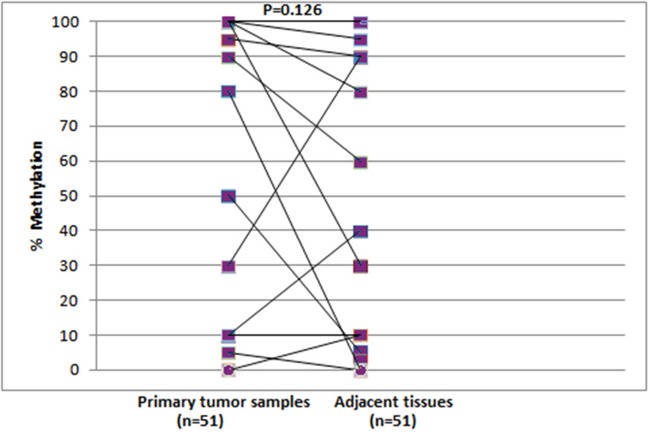
Comparison of *RASSF1A* promoter methylation levels in the paired primary tumor (n=51) and adjacent tissue (n=51) samples of group B, as estimated by MS-HRMA assay

We further evaluated the percentages of *RASSF1A* promoter methylation in primary tumor samples and adjacent tissues, by using the semi-quantitative MS-HRMA assay. *RASSF1A* promoter was found methylated in 27/67 (40.3%) primary tumor samples of group A and in 28/61 (45.9%) primary tumor samples of group B. 21/58 (36.2%) adjacent morphologically tumor cell-free tissues of group B were found methylated. The MS-HRMA assay can detect heterogeneous methylation; we found heterogeneously methylated samples both in group A (8/67, 11.9%) and in tumor samples of group B (7/61, 11.5%). We also observed heterogeneous methylation in 5/58 (8.6%) adjacent tissues of group B. According to the semi-quantitative MS-HRMA, in most positive cases *RASSF1A* promoter methylation was detected at a lower percentage in the adjacent morphologically tumor cell-free tissues, when compared to the paired primary tumors (Figure 2). However, there were three cases where the percentage of *RASSF1A* promoter methylation was higher in the adjacent tissue (Figure 2). No significant difference was observed (P=0.126, Mann-Whitney U test).

### Comparison between real-time MSP and MS-HRMA

When we compared our results derived for the same primary tumor samples in both group A and group B, by real-time MSP and MS-HRMA, the agreement between the two assays was almost perfect (Table 1). More specifically, in the group A, there was an agreement for 63/67 (94.0%) primary tumor samples (P<0.001, 2-sided Pearson χ^2^ test, k=0.876), while in the group B, there was an agreement for 58/61 (95.1%) samples (P<0.001, 2-sided Pearson χ^2^ test, k=0.900). In the group of adjacent tissue samples (group B), the agreement between real-time MSP and MS-HRMA was substantial (50/58, 86.2%, P<0.001, 2-sided Pearson χ^2^ test, k=0.689) (Table 1).

**Table 1:** Comparison between real-time MSP and MS-HRMA for *RASSF1A* promoter methylation in primary tumors (n=128) and adjacent tissues (n=58)

**Primary tumors: *RASSF1A* promoter methylation (group A, n=67)**			
**Real-time MSP**	**MS-HRMA**		**Total**
	**Unmethylated**	**Methylated**	
Unmethylated	38	2	40
Methylated	2	25	27 (40.3%)
Total	40	27 (40.3%)	67
Agreement (methods)	63/67 (94.0%), P<0.001, Cohen's kappa=0.876		
**Primary tumors**: ***RASSF1A*** **promoter methylation (group B, n=61)**			
**Real-time MSP**	**MS-HRMA**		**Total**
	**Unmethylated**	**Methylated**	
Unmethylated	33	3	36
Methylated	0	25	25 (41.0%)
Total	33	28 (45.9%)	61
Agreement (methods)	58/61 (95.1%), P<0.001, Cohen's kappa=0.900		
**Primary tumors**: ***RASSF1A*** **promoter methylation in both groups, (n=128)**			
**Real-time MSP**	**MS-HRMA**		**Total**
	**Unmethylated**	**Methylated**	
Unmethylated	71	5	76
Methylated	2	50	52
Total	73	55	128
Agreement (methods)	121/128 (94.5%), P<0.001, Cohen's kappa=0.888		
**Adjacent tissues**: ***RASSF1A*** **promoter methylation (group B, n=58)**			
**Real-time MSP**	**MS-HRMA**		**Total**
	**Unmethylated**	**Methylated**	
Unmethylated	35	6	41
Methylated	2	15	17 (29.3%)
Total	37	21 (36.2%)	58
Agreement (methods)	50/58 (86.2%), P<0.001, Cohen's kappa=0.689		

### Direct comparison of *RASSF1A* promoter methylation status in primary tumors, adjacent tissues and plasma ctDNA

We further directly compared *RASSF1A* promoter methylation status in 53 cases, where primary tumors, adjacent tissues and corresponding plasma ctDNA were available (triplets, n=53). *RASSF1A* promoter methylation status in primary tumors and adjacent tissues was evaluated using both real-time MSP and MS-HRMA, while in corresponding plasma samples only real-time MSP was used because of its higher sensitivity. In most cases there was a concordance between our findings in primary tumors, adjacent tissues and plasma (Table 2). In 45/53 (84.9%) cases we found an agreement for *RASSF1A* promoter methylation between primary tumor samples and adjacent tissues (P<0.001, 2-sided Pearson χ^2^ test, Cohen's kappa=0.674). According to the guidelines for the interpretation of k values, there is a substantial agreement between the two subgroups. In 33/53 (62.3%) cases we observed a slight agreement for *RASSF1A* promoter methylation between primary tumor samples and corresponding plasma, (P=0.227, 2-sided Pearson χ^2^ test, k=0.156) (Table 2). In group B, we used again the 53 triplets for the comparison between primary tumor samples and adjacent tissues using MS-HRMA. The agreement between the two subgroups was 47/53 (88.7%, P<0.001, 2-sided Pearson χ^2^ test, Cohen's kappa=0.768, substantial agreement).

**Table 2:** *RASSF1A* promoter methylation in primary tumors, adjacent tissues and corresponding plasma samples using real-time MSP (n=53, triplets)

**Primary tumors vs adjacent tissues: *RASSF1A* promoter methylation (n=53)**			
**Primary tumor**	**Adjacent tissue**		**Total**
	**Unmethylated**	**Methylated**	
Unmethylated	30	2	32
Methylated	6	15	21
Total	36	17	53
Agreement	45/53 (84.9%), P<0.001, Cohen's kappa=0.674		
**Primary tumors vs corresponding plasma**: ***RASSF1A*** **promoter methylation (n=53)**			
**Primary tumor**	**Corresponding plasma**		**Total**
	**Unmethylated**	**Methylated**	
Unmethylated	26	6	32
Methylated	14	7	21
Total	40	13	53
Agreement	33/53 (62.3%), P=0.227, Cohen's kappa=0.156		

**Figure 3:**
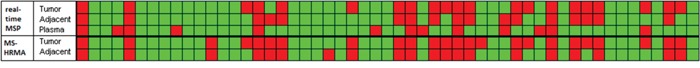
*RASSF1A* promoter methylation as evaluated both by real-time MSP and MS-HRMA, in group B: primary tumors, adjacent tissues and corresponding plasma samples (n=53) Red: positive sample (methylated), green: negative sample (unmethylated).

Our results on *RASSF1A* promoter methylation in primary tumors, adjacent tissues and corresponding plasma samples are shown in Figure 3. In six patients, *RASSF1A* promoter methylation was detected in the primary tumor and in the adjacent tissue by both assays and in corresponding cfDNA in plasma by real-time MSP. In five patients, *RASSF1A* promoter methylation was detected only in plasma, while the primary tumors and adjacent tissues were found negative by both assays.

### Prognostic significance of *RASSF1A* promoter methylation in HGSC

We further proceeded to the estimation of the clinical significance of *RASSF1A* promoter methylation status for the patients of group B, as Overall Survival (OS) and Progression-Free Survival (PFS) data were available along with other clinicopathological characteristics. The total number of patients is now different (n=47), because in some cases the clinical information was not available and all cases where OS≤4 months were excluded from the survival study. The median OS was 36 months while the median PFS was 12.5 months (starting date being the date of diagnosis; PFS was estimated based on the date of relapse; OS was estimated based on the date of death). The correlation between *RASSF1A* promoter methylation status of primary tumor samples with clinicopathological features of the patients is shown in Table 3. *RASSF1A* promoter methylation was significantly associated with tumor grade using both assays (real-time MSP: P=0.043, MS-HRMA: P=0.037) and regional lymph nodes (pN) using MS-HRMA (P=0.040). No significant correlations are found between *RASSF1A* methylation status of adjacent tissues and plasma samples, and clinicopathological characteristics (data not shown).

**Table 3:** Correlation of *RASSF1A* methylation status of primary tumor samples with clinicopathological features of the patients (group B)

Clinicopathological characteristics	*RASSF1A* promoter methylation (primary tumors, n=47)				
	n^a^	real-time MSP		MS-HRMA	
		% methylation	P-value (χ^2^ test)	% methylation	P-value (χ^2^ test)
**Age**					
≥ 64	24	9 (37.5)	0.908	11 (45.8)	0.642
< 64	23	9 (39.1)		9 (39.1)	
**Tumor grade (G)**					
G2	20	11 (55.0)	**0.043**	12 (60.0)	**0.037**
G3	27	7 (25.9)		8 (29.6)	
**Regional lymph nodes (pN)**					
N0	15	7 (46.7)	0.062^b^	8 (53.3)	**0.040**
N1	20	3 (15.0)		4 (20.0)	
**Distant metastasis (M)**					
M0	41	16 (39.0)	1	18 (43.9)	1
M1	6	2 (33.3)		2 (33.3)	
**Platinum resistance**					
Positive	8	2 (25.0)	0.697^b^	4 (50.0)	0.454^b^
Negative	34	12 (35.3)		12 (35.3)	
**Tumor rest**					
Positive	17	8 (47.1)	0.352	9 (52.9)	0.278
Negative	30	10 (33.3)		11 (36.7)	

^a:^ in cases where the total number of patients is different this is due to non-available clinical information

^b:^ Fisher's Exact Test

**Figure 4:**
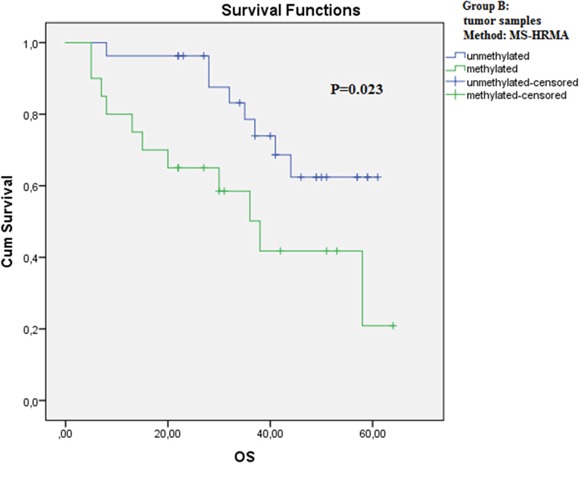
Kaplan-Meier estimates of overall survival (OS) for patients of group B with (green) or without (blue) *RASSF1A* promoter methylation in tumor FFPEs using MS-HRMA (P=0.023)

The Kaplan-Meier analysis was further performed to correlate OS and PFS data with *RASSF1A* promoter methylation status. In primary tumor samples, OS was found to be significantly correlated with *RASSF1A* promoter methylation status using MS-HRMA (P=0.023, log-rank test, Figure 4), whereas no significant correlation was observed using real-time MSP (P=0.157, log-rank test). No significant correlations were found between OS and *RASSF1A* promoter methylation status of adjacent tissues and plasma samples, and between PFS and *RASSF1A* promoter methylation status of all three subgroups (data not shown).

Finally, *RASSF1A* promoter methylation in primary tumor samples and all the available clinicopathological features were tested in univariate Cox Regression analysis for association with OS and PFS. *RASSF1A* promoter methylation status using MS-HRMA and platinum resistance were significantly associated with decreased OS (P=0.030 and P=0.019, respectively). The lack of correlation between OS and clinical parameters known to be of predictive value, like age and tumor rest, can be possibly explained by the relatively small cohort analyzed here. We next performed multivariate Cox Regression analysis for *RASSF1A* promoter methylation estimated with the MS-HRMA assay and platinum resistance in association with OS, but no independent prognostic significance was observed. The results are shown in detail in Table 4.

**Table 4:** Univariate and multivariate Cox Regression analysis between OS and univariate Cox Regression analysis between PFS, *RASSF1A* methylation status of primary tumor samples and clinicopathological features of the patients (group B)

**Univariate Cox Regression analysis (Dependent Variable: OS)**				
	**P-value**	**HR (hazard ratio)**	**95.0% CI for HR**	
			**Lower**	**Upper**
***RASSF1A*** **methylation (real-time MSP)**	0.166	1.896	0.767	4.688
***RASSF1A*** **methylation (MS-HRMA)**	**0.030**	**2.761**	1.102	6.915
**Age**	0.844	0.913	0.370	2.254
**Tumor grade (G)**	0.744	0.860	0.348	2.126
**Regional lymph nodes (pN)**	0.432	0.640	0.210	1.948
**Distant metastasis (M)**	0.784	1.189	0.345	4.096
**Platinum resistance**	**0.019**	**3.752**	1.245	11.306
**Tumor rest**	0.758	0.859	0.326	2.263
**Multivariate Cox Regression analysis (Dependent Variable: OS)**				
***RASSF1A*** **methylation (MS-HRMA)**	0.253	1.818	0.653	5.064
**Platinum resistance**	**0.024**	**3.588**	1.185	10.863
**Univariate Cox Regression analysis (Dependent Variable: PFS)**				
	**P-value**	**HR (hazard ratio)**	**95.0% CI for HR**	
			**Lower**	**Upper**
***RASSF1A*** **methylation (real-time MSP)**	0.943	1.029	0.468	2.264
***RASSF1A*** **methylation (MS-HRMA)**	0.682	1.179	0.536	2.596
**Age**	0.827	1.093	0.494	2.416
**Tumor grade (G)**	0.401	1.405	0.636	3.106
**Regional lymph nodes (pN)**	0.599	0.773	0.296	2.018
**Distant metastasis (M)**	0.797	0.851	0.249	2.909
**Platinum resistance**	0.403	-	-	-
**Tumor rest**	0.597	1.244	0.554	2.792

## DISCUSSION

*RASSF1A* promoter methylation is a common event in ovarian cancer, and was first identified in ovarian tumor samples over a decade ago [26–28]. Apart from primary tumors, benign cystadenomas and low malignant potential tumors exhibit *RASSF1A* promoter methylation as well [29, 30]. Choi et al. first tried to correlate *RASSF1A* promoter methylation with patients outcome, but did not find any correlation [30]. A possible explanation could be that in this study, all the ovarian cancer samples of serous histotype were concerned as a single cohort without taking into account the two subtypes, high- and low-grade serous ovarian cancer. However, it is now known that these two subtypes differ in the progenitor area and the tumors molecular profile [31]. High methylation frequency of *RASSF1A* has also been observed by Montavon et al. at HGSC tumor samples, however, the relatively small number of available survival data (n=37) could be a possible explanation for the lack of association between *RASSF1A* methylation and overall survival in this study [32]. Ibanez et al. screened ovarian tumors of different histology, with matched preoperative serum or plasma and peritoneal fluid samples for *RASSF1A* promoter methylation. They concluded for the first time that *RASSF1A* promoter methylation can be detected in cfDNA and represents an early event in ovarian carcinogenesis [33]. Other studies confirm the detection of methylated *RASSF1A* in plasma samples [34–36]. Bon Durant et al. compared *RASSF1A* promoter methylation between tumor and matched plasma, in 20 available sample pairs and observed 100% agreement. They also determined changes in *RASSF1A* methylation status during the course of treatment [34]. A phase II clinical trial has reported that demethylation of *RASSF1A* had a positive correlation with PFS indicating a possible role of *RASSF1A* promoter methylation in platinum resistance [37].

A large number of studies declare *RASSF1A* promoter methylation in the majority of human malignancies, including breast, endometrial and cervical cancer [23, 38]. Our group has shown the prognostic significance of *RASSF1A* promoter methylation in early stage breast cancer [39] and reported the frequent *RASSF1A* promoter methylation in cfDNA of operable gastric cancer patients [40]. Spitzwieser et al. investigated *RASSF1A* promoter methylation status in 17 breast cancer samples and their matched normal adjacent tissues using MS-HRMA and found high methylation frequencies in tumors and adjacent tissues, but no correlation between their methylation status [41]. A previous study also showed no significant concordance between methylation changes in 56 breast tumor and their paired adjacent normal tissues [42]. In endometrioid adenocarcinoma, Arafa et al. reported that *RASSF1A* promoter methylation is methylated in endometrial cancer samples but also in a small group of adjacent normal endometrium tissues surrounding the tumor [43]. Evaluation of *RASSF1A* promoter methylation in matched samples of ovarian cancer patients has been very limited so far. There is only one study, including 3 tumors and their matched normal adjacent tissues, where all 3 adjacent tissues were found non-methylated [28]. Promoter methylation in adjacent morphologically tumor cell-free tissues reflects field cancerization, also called field effect. Field effect describes all the genetic and epigenetic abnormalities found in adjacent tissues that are defined as morphologically normal [44].

**Figure 5:**
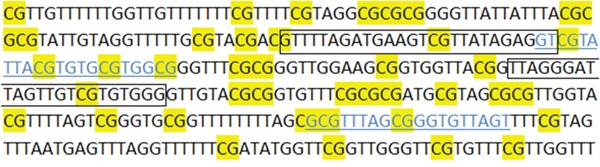
Primer sequences and positions of real-time MSP and MS-HRMA assays for *RASSF1A* promoter methylation The MSP primers are shown in blue underlined letters and the MS-HRMA primers are framed. The sequence is produced after SB conversion of gDNA. All CpGs are considered as methylated.

In the present study, we examined *RASSF1A* promoter methylation status in primary tumors, adjacent morphologically tumor cell-free tissues and corresponding plasma samples of patients with HGSC, using real-time MSP and MS-HRMA. The two assays showed almost perfect agreement when applied in the tumor samples of both groups, and substantial agreement in the adjacent tissues of group B. Two tumor samples from group A, 3 tumor and 6 adjacent samples from group B were found methylated with MS-HRMA and unmethylated with real-time MSP (Table 1). These discrepant results probably are due to the slight differences in the promoter region assessed by each assay (Figure 5). As shown in Figure 5, there is one extra CG in the forward MS-HRMA primer. When a sample is negative in real-time MSP, all CGs in MSP primers are unmethylated. But if the additional CG that is present only in the MS-HRMA primer is methylated in the sample, then the MS-HRMA result is expected to be positive. There were also 2 tumor samples from group A and 2 adjacent samples from group B where we observed methylation with real-time MSP, but not with MS-HRMA (Table 1). This is potentially due to the higher sensitivity of the real-time MSP assay compared to MS-HRMA.

In adjacent morphologically tumor cell-free tissues of group B, we observed rather high methylation levels using both MS-HRMA (36.2%) and real-time MSP (29.3%). This indicates field cancerization (field effect) and potentially cancer progression. We found no significant difference between tumor and adjacent tissue methylation level and in three cases, the percentage of *RASSF1A* promoter methylation was higher in the adjacent tissue (Figure 2). A potential explanation is the strong field effect that characterizes the cases studied, especially these three pairs.

According to our findings, *RASSF1A* promoter methylation is significantly correlated with OS when MS-HRMA is used (P=0.023), but no significant correlation is observed with real-time MSP (P=0.157). This fact gives an advantage to the MS-HRMA, although real-time MSP is a more sensitive assay. However, real-time MSP is preferable for methylation studies in plasma ctDNA, due to its higher sensitivity. In six samples, where the primary tumor was found unmethylated, the corresponding plasma samples were methylated. A potential explanation for this observation could be based on tumor heterogeneity; it is now clear that tissue biopsy represents a snapshot of tumor molecular profile, while cfDNA reflects the total genetic and epigenetic characteristics of a particular cancer. The effect of tumor heterogeneity in our results could be only shown if single cells analysis was performed. However we have not designed our study based on single cell analysis, this could be a nice idea for future studies. Moreover, cfDNA can originate not only from the primary tumor but from metastatic sites as well, from apoptotic and necrotic cells.

In conclusion, we performed a direct comparison study on *RASSF1A* promoter methylation in primary tumors, adjacent tissues and plasma samples in HGSC patients. We report for the first time that *RASSF1A* promoter is methylated in adjacent tissue surrounding the tumor in HGSC patients and that *RASSF1A* promoter methylation provides prognostic information since it is significantly correlated with OS. Our results indicate that the evaluation of *RASSF1A* methylation status in ovarian cancer has the potential to provide important prognostic information; however to verify this finding, more prospective studies should be performed. Taking into account that the primary tumor tissue is typically available only at primary diagnosis, it would be valuable to establish a non-invasive blood-based biomarker for stratifying response to platinum-based chemotherapy at primary diagnosis and for guiding individualized therapy decisions in the future.

## MATERIALS AND METHODS

### Clinical samples

Our study material consisted of two main groups of samples from patients with primary HGSC; a) group A that consists of 67 primary ovarian formalin fixed paraffin-embedded tissues (FFPEs) and b) group B that consists of 61 primary FFPEs, 58 available adjacent morphologically tumor cell-free tissues (FFPEs) and 59 available corresponding plasma samples (2mL). For the plasma sampling, two x 5ml ethylenediaminetetraacetic acid (EDTA) blood samples were collected at time point of diagnosis, before tumor surgery and before the application of therapeutic substances with an S-Monovette (Sarstedt AG & Co.). Blood was centrifuged at 1500g for 10min and the plasma supernatant was stored at -80°C until further usage. The available clinicopathological features for both groups are shown in Table 5. For the evaluation of the specificity of our assays, two groups of normal samples were recruited: a) a small group of 16 normal fallopian tube FFPEs that were obtained from women of the reproductive age group and b) a larger group of 51 plasma samples obtained from healthy women (2mL). All group A samples and the normal fallopian tube samples were obtained from the Pathology Department of IASO women's hospital, Athens, Greece. All group B samples were obtained from the Department of Pathology and the Department of Gynecology and Obstetrics, University Hospital of Essen, University of Duisburg-Essen, Germany. FFPE tissue blocks, retrieved from the Institute of Pathology and Neuropathology of the University Hospital of Essen, Germany were stained with Haematoxilin & Eosin and FFPE sections used for our assays were prepared and reviewed by a pathologist. We analyzed only samples with a tumor cell content of equal or more than 60%. All tissue samples were prepared under supervision of a pathologist. According to our pathologists, these are reasonable amounts of tumor tissue to study since 100% purity of tumor tissue can only be achieved in rare cases. All patients gave their informed written consent to participate in the study, which was approved by the Local Essen Research Ethics Committee (05/2856), and IASO women's hospital Ethics committee (Date: 05/2014). The OVCAR29 and IGROV1 ovarian cancer cell lines were used as positive controls in real-time MSP and MS-HRMA reactions for the detection of *RASSF1A* methylation.

**Table 5:** Available clinicopathological features of the patients

Clinicopathological characteristics	Group B(total n=64)n %	Group A(total n=67)n%
**Histology**		
Serous	64 (100)	67 (100)
**Tumor grade (G)**		
G1	2 (3.1)	-
G2	26 (40.6)	-
G3	36 (56.3)	67 (100)
**FIGO stage**		
I	1 (1.6)	13 (19.4)
II	2 (3.1)	38 (56.7)
III	39 (60.9)	12 (17.9)
IV	8 (12.5)	-
Unknown	14 (21.9)	4 (6.0)
**Age**	Median age=64	Median age=54
≥ median age	32 (50.0)	35 (52.2)
< median age	32 (50.0)	31 (46.3)
Unknown	-	1 (1.5)
**Regional lymph nodes (pN)**		
N0	18 (28.1)	
N1	29 (45.3)	
NX	4 (6.3)	
Unknown	13 (20.3)	
**Tumor (pT)**		
T1	6 (9.4)	
T2	6 (9.4)	
T3	52 (81.2)	
**Distant metastasis (M)**		
M0	55 (85.9)	
M1	8 (12.5)	
Unknown	1 (1.6)	
**Platinum resistance**		
Positive	10 (15.6)	
Negative	44 (68.8)	
Unknown	10 (15.6)	
**Tumor rest**		
Positive	25 (39.0)	
Negative	38 (59.4)	
Unknown	1 (1.6)	

### DNA isolation from FFPEs and plasma samples

Genomic DNA (gDNA) was isolated from FFPEs using the QIAamp^®^ DNA FFPE Tissue Kit 50 (Qiagen^®^, Germany) according to the manufacturer instructions. cfDNA from plasma (2mL) was extracted using the QIAamp^®^ Circulating Nucleic Acid kit 50 (Qiagen^®^, Germany), according to the manufacturer's instructions. DNA concentration was determined in the Nanodrop ND-1000 spectrophotometer (Nanodrop Technologies, USA).

### Sodium bisulfite conversion

1μg of gDNA and up to 0.5μg of cfDNA were chemically modified with sodium bisulfite (SB), in order to convert only the non-methylated cytosines to uracils, but not the methylated ones. SB conversion was performed with the EZ DNA Methylation-Gold™ Kit 200 (Zymo Research Corp., USA), according to the manufacturer's instructions. DNA was treated with the conversion reagent, incubated at 98°C for 10min and at 64°C for 2.5h. In each conversion reaction, dH_2_O and gDNA from OVCAR29 or IGROV1 ovarian cancer cell lines were used as negative and positive control, respectively. The Universal Methylated Human DNA Standard (Zymo Research Corp., USA) was used as fully methylated control. To evaluate the quality of SB converted DNA in all our samples, we used unmethylated *BRMS1* primers that are specifically designed to detect unmethylated *BRMS1* sequences after SB conversion, as previously described [20]. Real-time PCR amplification occurred in all SB converted DNA samples. The SB converted DNA was stored at -70°C until used.

### Real-time methylation specific PCR (real-time MSP)

We performed real-time MSP for the detection of *RASSF1A* promoter methylation, using specific primers adapted from a previous study [25]. The position of the primers in the promoter sequence is shown in Figure 5. 1μl of SB converted DNA was added in the PCR reaction mix, which consisted of 1X PCR buffer (Promega, USA), 2mM MgCl_2_ (Promega, USA), 0.2μM of each dNTP (Invitrogen, USA), 0.15μg/μL BSA (Sigma, Germany), 0.2μM of each primer (Integrated DNA Technologies, USA), 1X LC Green^®^ (Idaho Technology, USA) and 0.05U/μL GoTaq^®^ DNA polymerase (Promega, USA). dH_2_O was added to a final volume of 10μL. Protocol conditions were: 1 cycle at 95°C for 2min, followed by 45 cycles of: 95°C for 10s, 65°C for 15s and 72°C for 20s, and a final cooling cycle at 40°C for 30s. All real-time MSP reactions were performed in the LightCycler^®^ 1.5 instrument (Roche Applied Science, Germany).

### Methylation-sensitive high-resolution melting analysis (MS-HRMA)

**Figure 6:**
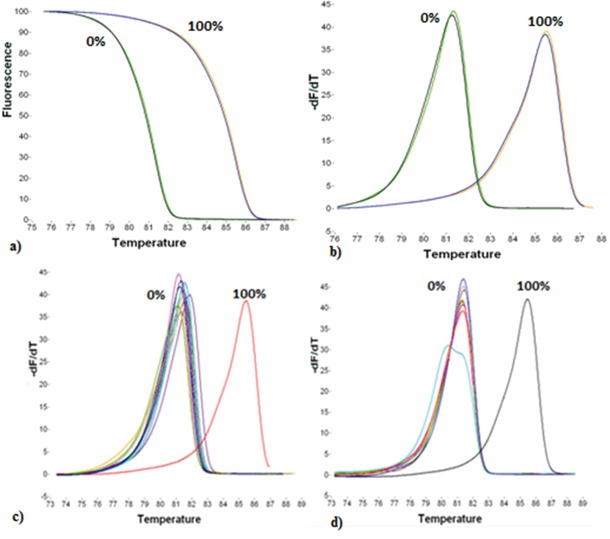
Analytical specificity and reproducibility of the MS-HRMA assay **a**. Normalized melting curves of the fully non-methylated (0%) and the fully methylated (100%) control. **b**. Derivative plots of the 0% and 100% methylated controls. **c**. Derivative plots of the 16 normal fallopian tube FFPEs (0%). **d**. Derivative plots of normal plasma samples from healthy women (0%).

For the semi-quantitative estimation of *RASSF1A* promoter methylation, we used specific primers adapted from a previous study [45]. The position of the primers in the promoter sequence is also shown in Figure 5. In this assay, methylation independent (MIP) primers allow for the equal amplification of both methylated and non-methylated target sequences. 1μl of SB converted DNA was added in the PCR reaction mix, which consisted of 1X PCR buffer (Promega, USA), 2.5mM MgCl_2_ (Promega, USA), 0.2μM of each dNTP (Invitrogen, USA), 0.25μg/μL BSA (Sigma, Germany), 0.25μM of each primer (Integrated DNA Technologies, USA), 1X LC Green^®^ (Idaho Technology, USA) and 0.05U/μL GoTaq^®^ DNA polymerase (Promega, USA). dH_2_O was added to a final volume of 10μL. The initial real-time PCR protocol conditions were: 1 cycle at 95°C for 2min, followed by 50 cycles of: 95°C for 10s, 63°C for 15s and 72°C for 20s, and a final cooling cycle at 40°C for 30s. All reactions were performed in the LightCycler^®^ 1.5 instrument (Roche Applied Science, Germany). After PCR amplification, MS-HRMA was performed in the HR-1 High Resolution Melter instrument (Idaho Technology, USA). Melting data acquisition began at 69°C and ended at 95°C, with a ramp rate of 0.30°C/s. After melting transition, fluorescence data normalization was performed, so that the four (1-4) vertical cursors of the instrument software are positioned in new adjusted temperatures in the same numeric order, from left to right. At the ramp rate of 0.30°C/s, a temperature range of 0.5°C was set between each cursor pair. Finally, the derivative plots were displayed in order to compare each sample's melting peak with those of the controls and have the semi-quantitative estimation of the methylation level. The totally methylated and non-methylated products have a melting temperature (T_m_) of 86°C and 81°C, respectively (Figure 6a, 6b).

### Analytical validation of the assays

#### Analytical specificity

We first verified that unconverted gDNA was not detected. The Universal Methylated Human DNA Standard (100% methylated control) was used as the fully methylated positive control in both assays. We also checked *RASSF1A* promoter methylation status of OVCAR29 and IGROV1 cell lines. Both cell lines were found methylated by using the real-time MSP assay and their melting curves resembled to those of the 100% positive control, according to the MS-HRMA assay. We did not use human placental gDNA as a fully non-methylated control, as it is reported that *RASSF1A* promoter is methylated in placental DNA [46], a fact that we also verified by both real-time MSP and MS-HRMA. We have used normal fallopian tube FFPE samples, as fully non-methylated controls, since unmethylated reference DNA from any tissue in which the target sequence does not show methylation, can be used as a source of unmethylated reference [47]. According to our results, both real-time MSP and MS-HRMA assays were highly specific, since *RASSF1A* promoter methylation was not detected at all both in the small group of fallopian tube FFPEs (0/16, 0%) and in the group of plasma samples from healthy women (0/51, 0%) (Figure 6c, 6d).

#### Analytical sensitivity

**Figure 7:**
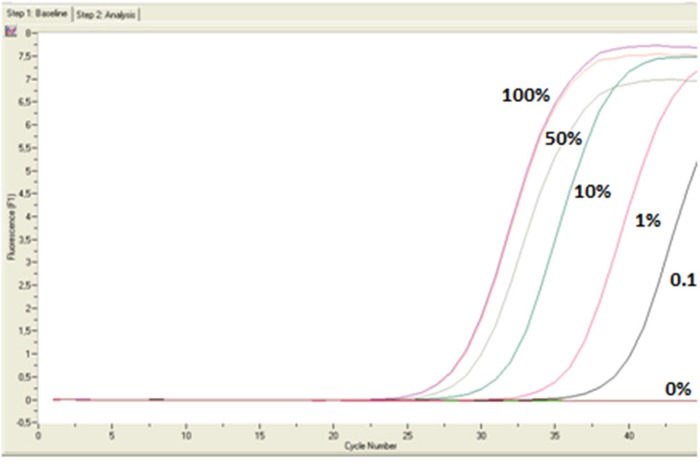
Analytical specificity and sensitivity of real-time MSP assay determined by the use of the dilutions (0%, 0.1%, 10%, 50%, 100%)

**Figure 8:**
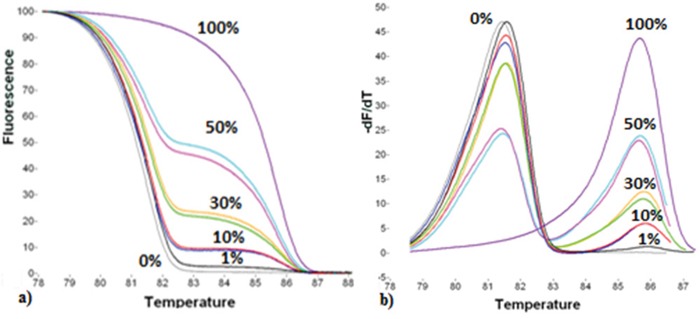
Analytical sensitivity and reproducibility of the MS-HRMA assay **a**. Normalized melting curves of the dilutions (0%, 1%, 10%, 30%, 50%, 100%). **b**. Derivative plots of the same dilutions.

To estimate the analytical sensitivity of real-time MSP and MS-HRMA assays, we prepared synthetic standards by mixing one fully non-methylated DNA sample with the OVCAR29 cell line; we prepared serial dilutions: 0%, 0.1%, 1%, 10%, 30%, 50% and 100% for both assays. According to our results, real-time MSP assay detects down to 0.1% of *RASSF1A* promoter methylation in the presence of 99.9% non-methylated sequences (Figure 7), while MS-HRMA detects down to 1% of *RASSF1A* promoter methylation in the presence of 99% non-methylated sequence (Figure 8).

### Statistical analysis

To estimate the agreement between the two assays in each sample group and the correlation of methylation status between subgroups of the group B, we calculated Pearson χ^2^ and Cohen's Kappa coefficient. P values < 0.05 were considered statistically significant. The k values were interpreted according to the guidelines. The Kaplan-Meier method was used for the calculation of OS and PFS curves and log-rank test was performed for the comparisons. Cox regression analysis was also performed for the estimation of hazard ratio. All statistical analysis was performed by using the SPSS Windows version 22.0 (SPSS Inc., Chicago, IL).
